# Simplifying perovskite solar cell fabrication for materials testing: how to use unetched substrates with the aid of a three-dimensionally printed cell holder

**DOI:** 10.1098/rsos.241012

**Published:** 2024-09-11

**Authors:** Joaquín Valdez García, Mahboubeh Hadadian, Rustem Nizamov, Paavo Mäkinen, Noora Lamminen, Paola Vivo, Kati Miettunen

**Affiliations:** ^1^ Department of Mechanical and Materials Engineering, Faculty of Technology, University of Turku, Vesilinnantie 5, Turku FI-20500, Finland; ^2^ Hybrd Solar Cells, Faculty of Engineering and Natural Sciences, Tampere University, P. O. Box 541, Tampere FI 33014, Finland

**Keywords:** perovskite solar cells, TCO patterning, IV characterization, 3D printing

## Abstract

This work demonstrates that unetched substrates can be reliably used in perovskite solar cell (PSC) fabrication. Chemical etching and laser patterning of the bottom electrodes are time- and resource-consuming processes. In particular, when testing novel conductive substrate materials, such as metallic or bio-based substrates, etching or patterning could be entirely unfeasible or could require significant process optimization. Avoiding these steps could accelerate research on PSCs, yet the literature shows no attempts to override these steps. Here, PSCs were fabricated and characterized using three-dimensionally printed holders with spring-loaded pins. We show that devices made on unetched substrates have, on average, a similar performance to those made on etched substrates (16 ± 1% and 16.0 ± 0.7%, respectively). Our study provides a new strategy for fabricating PSCs, particularly when etching and laser patterning are impractical.

## 1. Introduction

Perovskite solar cells (PSCs) are emerging as a promising technology for energy harvesting. They have attracted much attention due to their constantly increasing power conversion efficiency (*ƞ*) and their ease of fabrication. The thinness of their active layer and the low temperature used in their fabrication have made them suitable for deposition on substrates other than glass, such as synthetic polymers (e.g. PET and PEN) [1–4], titanium and steel foil [5,6] and even paper and wood [7–9]. New materials and fabrication processes are constantly being tested to increase their stability and efficiency; however, their effectiveness requires proof based on the characterization of each of the many new devices.

The laboratory-scale fabrication of PSCs tends to be performed on substrates smaller than 10 cm^2^. Efficient use of the substrates is usually accomplished by generally aiming to fit as many pixels as possible, but this is done at the expense of using complicated patterns on the bottom electrode. The removal of indium tin oxide (ITO) and fluorine-doped tin oxide (FTO) via chemical etching or laser patterning are standard procedures, but this removal process requires time and resources and therefore represents a hindrance to experimental PSC research. Etching and patterning are also used to reduce the losses related to sheet resistance in a large-area device by dividing it into smaller pieces [10,11], but the improvement these processes offer on a research scale is negligible and only complicates the fabrication process [12]. Furthermore, etching and laser patterning are unfeasible when the substrate is inherently conductive, as is the case with metallic substrates, or where the electrode is not a layer on top of the substrate but is embedded in a flexible substrate [13,14]. Consequently, the current characterization techniques, which are limited to rigid and etchable substrates, create the risk of disregarding alternative substrate materials in PSC research.

Developing an etching process for each possible new substrate material is also laborious, and avoiding that step would allow ready testing of a wide range of materials by eliminating the complications of an etching or patterning process from the screening stage. Furthermore, from an eco-design perspective, depositing ITO only to remove it later is environmentally incongruent with the goal of sustainable energy production. ITO is the most expensive and environmentally impactful part of a perovskite module, representing 56% of its cost and 96% of its carbon footprint [15], while FTO constitutes around 58% of the cost [16] and 40% of the carbon footprint embedded in the materials of typical FTO/TiO_2_ PSCs [17]. These costs have spurred research into the reuse of FTO and ITO substrates [15,18], and studies have now demonstrated that the performance of devices made on recycled substrates can even surpass those made on pristine ones [19]. Nevertheless, the eventual recycling of solar devices would favour unetched substrates, since reusing patterned substrates requires keeping the previous device geometry [20]. A more sustainable approach is to remove the layers that have degraded and redeposit new ones on top of a working electrode, rather than recovering all the materials at an elemental level [21].

A current belief in the PSC field is that an intrinsic need exists for etching the substrate to avoid short-circuiting the devices. Indeed, this is truly a requirement to avoid short circuits when the characterization instruments pose a risk of scratching the layers. For this reason, the top electrode is extended to an area where the bottom electrode has been removed. However, if both electrodes can be accessed directly without scratching the device, etching and patterning would be unnecessary. In this paper, we demonstrate how fused deposition moulding (FDM) three-dimensional printed holders with spring-loaded pins (commonly known as ‘pogo pins’) can be used to create cells made on etched or unetched FTO substrates with practically equal performance. These findings should help researchers reduce the time and resources expended when optimizing their devices and facilitate the fabrication of PSCs where chemical etching and laser patterning are unviable.

## 2. Material and methods

**Figure 1.**
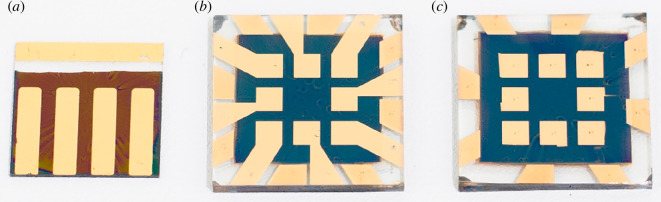
Devices from four-pixel group (*a*), eight-pixel etched group (*b*) and eight-pixel unetched group (*c*). Etched devices in the four-pixel group had part of the FTO chemically etched, while the FTO in etched devices of the eight-pixel group devices was patterned with a laser.

The prepared samples were divided into two groups based on the patterning of their bottom electrode and their pixel distribution. The bottom electrode of the four-pixel group was chemically etched and each device had four pixels. The bottom electrode of the eight-pixel group was laser patterned and each device had eight pixels. A device from each group is depicted in figure 1.

### 2.1. PSC preparation

FTO substrates for the four-pixel group (20 × 20 × 2 mm, TEC15) and unetched substrates for the eight-pixel group (25.4 × 25.4 × 2.2 mm, TEC15) were purchased from Greatcell Solar. Laser-patterned FTO substrates of the eight-pixel group were purchased from OPV Tech (25.4 × 25.4 × 2.2 mm, TEC15). The FTO patterns of both groups are provided in the electronic supplementary material, figure S1.

Half of the substrates in the four-pixel group were partially covered with Kapton^®^ tape, and only a part of the electrode was etched using zinc powder (Sigma Aldrich) and a 4M hydrochloric acid solution (Sigma Aldrich). Half of the substrates used in the eight-pixel group were pre-patterned by the supplier.

The four-pixel group substrates were first cleaned thoroughly with a toothbrush and a 2% Hellmanex™ solution in deionized water. After a rinse with deionized water, the substrates were cleaned in an ultrasonication bath in a 2% Hellmanex™ in deionized water solution for 15 min. They were thoroughly rinsed with deionized water and ultrasonicated again in acetone for 15 min. After thorough rinsing with acetone, the substrates were ultrasonicated in isopropanol for another 15 min, rinsed with isopropanol, and then dried with a nitrogen gun. The eight-pixel group substrates were cleaned in a similar fashion but using Mucasol instead of Hellmanex™.

The four-pixel group substrates were spray coated with a mixture of 0.16M titanium diisopropoxide bis(acetylacetonate) (Sigma Aldrich) and 0.4M acetylacetone (Sigma Aldrich) in absolute ethanol (Altia, 99.5%) to produce a layer of compact TiO_2_ (c-TiO_2_). The substrates were kept at 460°C on a hot plate and sprayed with the solution with an airbrush and compressed air at an air pressure of 1.5 bar from a distance of 30 cm and at a 45° angle. A glass piece was placed on a segment of the substrates to avoid the deposition of a TiO_2_ layer on that segment. The substrates were left to slowly cool down to room temperature (approx. 3 h) to avoid glass breakage. A mesoporous TiO_2_ layer (meso-TiO_2_) was subsequently produced by preparing a 150 mg ml^−1^ TiO_2_ paste (Greatcell Solar, 30 NR-D) in absolute ethanol. A piece of Kapton^®^ tape was attached to the substrates to protect the FTO from being covered with TiO_2_, and the solution was then spin coated for 10 s at 4000 rpm. The Kapton^®^ tape was removed, and the substrates were kept on a hot plate at 100°C for 10 min, followed by sintering at 450°C for 30 min.

The eight-pixel group c-TiO_2_ electron transport layer (ETL) was made by spraying a 17.3 vol% solution of titanium diisopropoxide bis(acetylacetonate) (Sigma Aldrich) in 2-propanol at 450°C. At least 12 layers of the solution were sprayed onto the substrates and left to anneal at 450°C for at least 45 min. The meso-TiO_2_ layer for the eight-pixel group was then made in a similar fashion to that used for the four-pixel group.

The perovskite layer for both groups was prepared by first making a 4 : 1 solution of dimethylformamide (DMF) (Sigma Aldrich, 99.8% anhydrous) and dimethyl sulfoxide (DMSO) (Sigma Aldrich, 99.9% anhydrous). Next, 1.5 M solutions of PbI_2_ (TCI, >98% perovskite grade) and PbBr_2_ (TCI, >98% perovskite grade) were prepared using the DMF : DMSO solution. A 1.5 M CsI (Abcr) solution was made in DMSO. Formamidinium iodide (FAI) (Greatcell Solar, >99.99%) and methylammonium bromide powder (MABr) (Greatcell Solar, >99.99%) were then mixed with PbI_2_ and PbBr_2_, respectively, to obtain FAPbI_3_ and MAPbBr_3_. These perovskite solutions were mixed with CsI to obtain a Cs_0.05_(FA_0.83_MA_0.17_)_0.95_Pb(I_0.83_Br_0.17_)_3_ (CsFAMA) precursor solution. Finally, the perovskite layer was deposited by spin coating onto the prepared substrates by dropping (50 µl drops). The substrate was first spun at 1000 rpm for 10 s and then at 6000 rpm for 20 s. This was followed by spin coating 200 µl of chlorobenzene (Sigma Aldrich) onto the substrate 5 s before the end of the second step. The substrates were then placed on a hot plate at 100°C for 1 h. All steps in this process were conducted inside a glovebox with a N_2_ atmosphere.

For the four-pixel group hole transport layer (HTL), a 70 mM spiro-MeOTAD (Lumtec) solution in chlorobenzene was prepared and doped with 4-*tert*-butylpyridine (tBP, Sigma Aldrich), lithium bis(trifluoromethanesulphonyl)imide (LiTFSI, Sigma Aldrich) and tris(2-(1H-pyrazol−1-yl)−4-*tert*-butylpyridine) cobalt (III) tris-(bis(tri-fluoromethane) sulphonimide) (FK209, Dyenamo). (The molar equivalents to spiro-MeOTAD were 3.3, 0.5 and 0.05, respectively). This solution was dynamically spin coated onto the substrates for 10 s at 4000 rpm. The devices were then placed in a sample box covered in aluminium foil inside a desiccator overnight to oxidize the spiro-MeOTAD layer without absorbing moisture. The eight-pixel group HTL was dynamically spin coated by dropping 80 µl of a 29.5 mM spiro-MeOTAD solution at 1800 rpm for 30 s. The same dopants were added but at different molar concentrations. (The tBP, LiTFSI and FK209 molar equivalents to spiro-MeOTAD were 3.2, 0.53 and 0.1, respectively). Afterwards, the cells were removed from the glovebox and placed in a dry box overnight to oxidize the spiro-MeOTAD.

The bottom electrodes of the four-pixel group were accessed by removing the perovskite layer and HTL by scratching them with a scalpel and acetonitrile. Finally, an 80-nm gold layer was evaporated onto the tops of the devices as the counter electrode. The perovskite and HTL of the eight-pixel group were removed by wiping with DMSO and a 100-nm gold layer was used as the top electrode.

### 2.2. Holder design and fabrication

**Figure 2.**
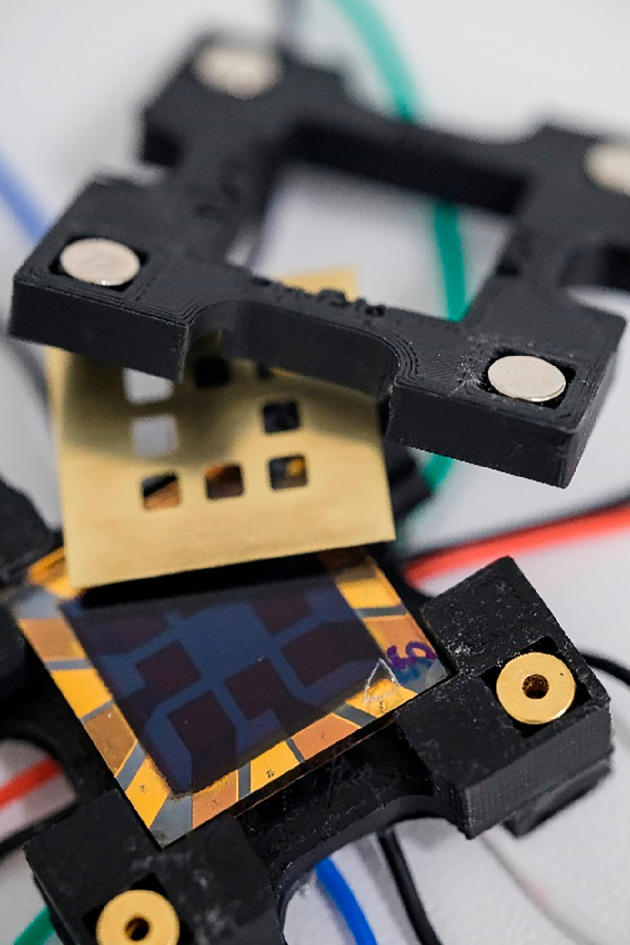
(Bottom to top) PSC three-dimensionally printed holder base and cables, eight-pixel PSC, brass characterization mask and three-dimensionally printed holder lid with magnets.

The holder for PSC characterization was designed and fabricated using a Prusa MK3S + three-dimensional printer equipped with a 0.4-mm stainless steel nozzle. The printing was done inside an Original Prusa Enclosure to maintain a stable thermal environment, which is crucial for high-quality three-dimensional prints (figure 2).

A satin powder-coated sheet was utilized for the printing, with a black EasyABS filament chosen as the printing material for its superior mechanical and thermal properties. The print settings were adjusted to a layer height of 0.1 mm to achieve the desired resolution in the three-dimensionally printed holder.

Harwin P70−1020045R spring-loaded pins were inserted into the base of the holder to contact the PSC electrode. To lessen the force applied to the cell, pogo pins were chosen because they allow for pressure against the cells, while their springs limit this pressure. Copper cables were soldered to the back side of the pins to contact the characterization instruments (electronic supplementary material, figure S2). To secure the PSCs in position, a lid was constructed with neodymium magnets at the corners of the holder. The force of the magnets was mainly applied to the corners, so the pogo pins were not fully engaged.

More detailed schematics are provided in electronic supplementary material, figures S3 and S4, together with the three-dimensional models for printing.

### 2.3. Device characterization

The current density–voltage curves (JV curves) for the four-pixel group were obtained using a Newport Oriel Instruments model 92 250A solar simulator, and a Keithley 2636 source measure unit (SMU) under 1 sun illumination (100 mW cm^−2^ AM 1.5G) and a 50 mV s^−1^ scan rate in both forward and reverse scan directions. A brass mask with a 12 mm^2^ aperture was used. The JV curves for the eight-pixel group were obtained using a Wavelabs Sinus−70 solar simulator and a Keithley 2450 SMU under one sun illumination, a 50 mV s^−1^ scan rate, and a brass mask with a 10 mm^2^ aperture. The JV curves were smoothed in Origin 2016.

Charge transfer in the devices was studied using electrochemical impedance spectroscopy (EIS). Measurements were performed at open-circuit voltage under 1 sun illumination using a Peccell PEC-L01 solar simulator and a Gamry Reference 600+potentiostat from 10^−1^ to 10^6^ Hz. Data were analysed using the BioLogic EC-Lab^®^ V11.52 software.

## 3. Results and discussion

**Figure 3.**
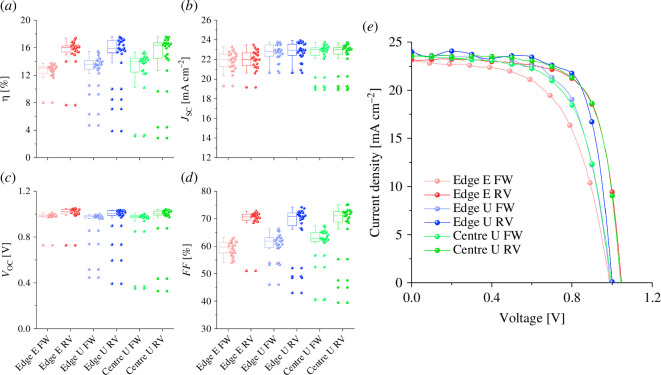
Performance of the etched (*e*) and unetched (*u*) devices of the four-pixel group in forward (FW) and reverse (RV) scan directions measured at the edge of the cell (edge) and in the centre (centre). (*a*) Power conversion efficiency, (*b*) short-circuit current density, (*c*) open-circuit voltage, and (*d*) fill factor of all devices and (*e*) JV curves of the champion devices.

Generally, extensions of the top electrode are deposited on top of regions where the bottom electrode has been removed. Typically, a metallic paste (commonly silver) is applied onto the electrodes to improve the contact with the characterization instruments. Because the paste penetrates all the layers of the device until it reaches the substrate, it can potentially short-circuit the device if it contacts the bottom electrode. Spring-loaded pins enable measurement of the devices without the need to etch the bottom electrode or apply any paste. This was confirmed by fabricating a custom three-dimensionally printed holder with spring-loaded pins to measure a group of 12 four-pixel cells. Six of them were made on unetched substrates and the other six were made on substrates where part of the FTO was chemically removed. For a fair comparison, the four-pixel group cells were all measured using the same holder, with the pins contacting the electrode in the same position, away from the illuminated area on the edge of the cell. As shown in figure 3, the difference between etched and unetched devices was negligible and the average performance of all devices was very similar. The power conversion efficiency (*η*) of unetched devices measured from the edge of the cell (edge U) was practically identical (16 ± 1%) to that of the etched ones (edge E) (16.0 ± 0.7%). The short-circuit current density (*J*_SC_) was higher for the edge U devices (22.9 ± 0.8 mA cm^−2^) than for the edge E devices (22.1 ± 0.8 mA cm^−2^), as indicated in table 1. This could be due to the FTO in the edge region of the cell. As the other layers stack on top of the FTO, a more uniform gold electrode can form. On the etched devices, a boundary between the edge of the cell and the centre can create defects in the gold electrode, thereby reducing the extracted current.

**Table 1.** Average performance parameters for reverse scan direction and their standard deviation. Outlaying values were omitted.

device	number of pixels	*η* (%)	*J_SC_* (mA cm^−2^)	*V*_OC_ (V)	FF (%)
four-pixel group					
edge etched	24	16.0 ± 0.7	22.1 ± 0.8	1.02 ± 0.03	71 ± 2
edge unetched	24	16 ± 1	22.9 ± 0.8	1.01 ± 0.02	71 ± 3
centre unetched	24	16 ± 2	23.1 ± 0.4	1.01 ± 0.02	72 ± 3
eight-pixel group					
edge etched	47	14 ± 2	18.7 ± 0.9	1.12 ± 0.02	68 ± 5
centre unetched	48	15 ± 2	19 ± 2	1.11 ± 0.02	72 ± 4

Compared with measurements made under the active area, a longer distance exists between the point of contact and the illuminated area when measuring from the edges of the cell, and this would result in higher series resistance and a consequent decrease in performance. This was verified by constructing another three-dimensionally printed holder with pins that contacted the pixels from the centre of the cell. The same unetched devices were then measured with this new holder (centre U). Compared with the measurements from the edge of the cell (edge U), the centre U results showed a very similar *η* (16 ± 2% and 16 ± 1%, respectively). Overall, the average performance was practically identical, proving that etching is unnecessary at this scale.

**Figure 4.**
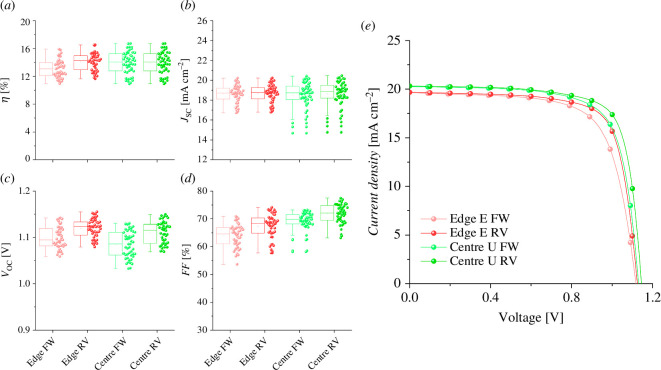
Performance of the etched (*e*) and unetched (*u*) devices of the eight-pixel group in forward (FW) and reverse (RV) scan directions measured at the edge of the cell (edge) and in the centre (centre). (*a*) Power conversion efficiency, (*b*) short-circuit current density, (*c*) open-circuit voltage, (*d*) fill factor, (*e*) JV curves of champion devices.

Another group of cells was then fabricated to verify whether these results could be transferred to a more complicated pixel distribution. The top electrode of the cells made on etched substrates was deposited from the centre of the cell to the edge (figure 1, middle). To make devices on unetched substrates, this design had to be modified to avoid having the gold top electrode touch the FTO. Therefore, a part of the gold evaporation mask was covered during deposition (figure 1, right). The eight-pixel group cells showed a higher difference in *ƞ* between etched and unetched devices due to a small but noticeable increase in FF (from 68 ± 5% to 72 ± 4%, respectively) (figure 4). This difference can be studied by performing EIS on the devices to obtain their series resistance (*R*_S_).

**Figure 5.**
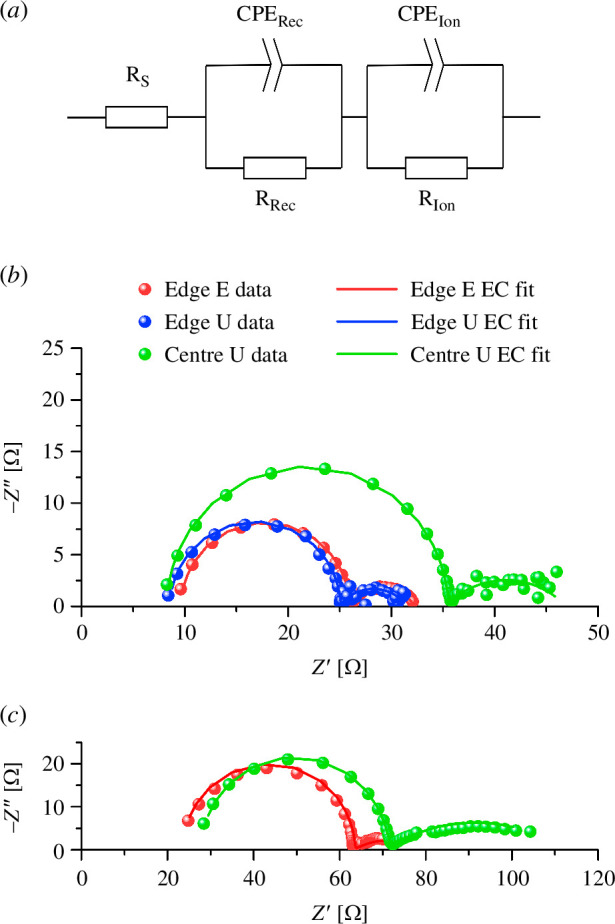
(*a*) EC used for EIS. (*b*) The four-pixel group Nyquist plots of champion devices. (*c*) The eight-pixel group Nyquist plots of champion devices.

EIS characterization was performed on several devices to further understand how charge transport differs in each kind of cell. EIS is done by measuring the electrical impedance of the device while injecting a low amplitude AC voltage of varying frequency over a steady DC voltage. In a Nyquist plot, the impedance data are represented by resistance on the *X*-axis (the real part) and impedance on the *Y*-axis (the imaginary part). The electrochemical processes in PSCs during EIS resemble the charge and discharge behaviour of an RC circuit, and this appears in the Nyquist plot as a semicircle. Due to the range of frequencies used in EIS, two semicircles usually appear for single-junction PSCs. Unlike Bode plots, Nyquist plots do not show frequency values; however, high-frequency values are generally on the left side of the plot and low-frequency values on the right. In this work, we based our EIS analysis on the study made by Roose *et al.* [22]. As shown in figure 5*a*, the behaviour of a PSC during EIS can be represented as a circuit, starting with a resistive element followed by two R-CPE (resistor-constant phase element) sections. Each part of the equivalent circuit (EC) is represented in the Nyquist plot. The first resistive element is *R*_S_ and it refers to the real ohmic resistance the current experiences in the electrodes (mainly due to resistance in the FTO layer) until it reaches the measuring equipment. Since *R*_S_ is purely resistive, it only has an *X* component, and its value corresponds to the distance between 0 and the start of the first semicircle. The first semicircle corresponds to the high-frequency recombination processes in the cell; it has a real resistive value (*R*_Rec_) and is equal to the diameter. *R*_Rec_ has an exponential dependence on the voltage; thus, even small changes in *V*_OC_ have large impacts [23]. The capacitive elements in the R-CPE segment have a frequency-dependent behaviour, and these create the shapes that are characteristic of Nyquist plots. For a certain range of frequencies, the CPE opposes the current, such as a capacitor. This appears as an increase in the imaginary part, and it is what starts to form the semicircle. CPE_Rec_ is present at high frequencies and is attributed to recombination processes inside the PSC (except for radiative recombination, which is too fast to observe with EIS) [24] and the geometric capacitance of the device. A second semicircle appears at low frequencies, and this can also be represented by another R-CPE segment in the EC. The frequencies in this range correspond to ionic motion interactions with the AC wave inside the PSC [22]. EIS offers complementary insight to understand how charge transport affects the PSC performance. *R*_S_ is the result of real resistances inside the cell (i.e. the resistance of the active material, a resistance at the interface between different layers and the resistance of the electrode). Since *R*_S_ is an inherent characteristic of the cell and is independent of the voltage, it impacts FF and, through that, *ƞ* as well. High charge recombination negatively affects the overall performance of the cell. As charges recombine inside the cell, this lowers *V*_OC_ and *J*_SC_ and results in a low *R*_Rec_. Finally, a high *R*_Ion_ indicates slow ion mobility, resulting in high hysteresis. Ideally, a good device should have low *R*_S_ and *R*_*Ion*_*,* but a high *R*_Rec_.

The four-pixel group centre U and edge U devices exhibited higher FF, and consequently, a higher *ƞ*, than was observed for edge *E*. This could be explained by their lower *R*_S_, as shown in table 2. Nonetheless, the differences are too small to be significant, proving how etched substrates can be substituted with unetched ones at this scale. Additionally, edge *E* shows almost no difference in *R*_S_ when measured from the centre of the device (electronic supplementary material, figure S5). By contrast, the eight-pixel group devices showed some differences between edge *E* and centre *U* devices. The largest difference between them lies in a higher FF in centre U cells—a product of their low *R*_S_. This difference in FF confirms our hypothesis that moving the contact point directly under the illuminated area in the cell increases the performance.

**Table 2.** Average impedance parameters and s.d. of different devices by EIS. Values are taken from their EC fit.

device	pixels considered	*R_S_* (Ω)	*R*_Rec_ (Ω)	*CPE*_Rec_ (nF)	*R*_Ion_ (Ω)	*CPE*_Ion_ (mF)
four-pixel group						
edge etched	8	11 ± 2	18 ± 4	66 ± 14	7 ± 2	15 ± 9
edge unetched	7	10 ± 2	15 ± 3	86 ± 9	6 ± 2	18 ± 6
centre unetched	7	9 ± 1	23 ± 6	67 ± 7	10 ± 4	13 ± 2
eight-pixel group						
edge etched	13	31 ± 10	84 ± 43	117 ± 300	37 ± 11	13 ± 21
centre unetched	13	14 ± 6	60 ± 11	20 ± 2	57 ± 15	11 ± 14

## 4. Conclusions

This work proves that unetched substrates can be used in PSCs with the help of three-dimensionally printed plastic measurement holders. Two groups of PSCs with an FTO/c-TiO_2_ and meso-TiO_2_/CsFAMA/spiro-MeOTAD/gold structure and different pixel distributions were fabricated and characterized. The devices were fabricated on unetched and etched substrates for comparison. JV curves showed that devices made on unetched and etched substrates exhibited similar efficiencies (16 ± 1% and 16.0 ± 0.7%, respectively). The ease of customization of three-dimensionally printed holders enabled us to compare the performance of the same devices when the holder contacts the electrode directly underneath the illuminated area. JV curves showed the similarity in the efficiency of the devices measured underneath the illuminated area and away from it (16 ± 2% and 16 ± 1% on average, respectively). Furthermore, impedance spectroscopy allowed us to explain how charge transport influences these small differences. Overall, we demonstrate the potential use of unetched substrates on a research scale and provide an alternative fabrication option when etching and laser patterning are unfeasible.

## Data Availability

The datasets supporting this article have been uploaded as part of the electronic supplementary material [25].
